# Carrier thermometry of cold ytterbium atoms in an optical lattice clock

**DOI:** 10.1038/s41598-018-26367-8

**Published:** 2018-05-21

**Authors:** Chengyin Han, Min Zhou, Xiaohang Zhang, Qi Gao, Yilin Xu, Shangyan Li, Shuang Zhang, Xinye Xu

**Affiliations:** State Key Laboratory of Precision Spectroscopy, East China Normal University, Shanghai, 200062 China

## Abstract

The ultracold atomic gas serving as the quantum reference is a key part of an optical lattice clock, and the temperature of atoms in the optical lattice affects the uncertainty and instability of the optical lattice clocks. Since the carrier spectrum of the clock transition in the lattices reflects the thermal dynamics of cold atoms, the temperature of atoms can be extracted from the carrier spectrum in a non-magic wavelength lattice of ytterbium optical clocks. Furthermore, the temperatures obtained from the carrier spectra are in good agreement with the results obtained by the time-of-flight method and thermometry based on the sideband spectrum. In addition, the heating effects caused by the lattice laser are studied on the basis of the sample temperatures.

## Introduction

In the last decade, research in the field of optical lattice clocks has experienced a fast development, especially the clocks based on cold ^87^Sr atoms and ^171^Yb atoms^1–9^, which both surpass the performance of the best ^133^Cs primary standards^10,11^. The best ^87^Sr clock now achieves an instability of 2.2 × 10^−16^ at 1 s and a total uncertainty of 2.1 × 10^−18^ in fractional frequency units^12^. These state-of-the-art clocks benefit from the great control of ultracold atoms that play the role of quantum references. They are confined in tight optical lattices with the ac Stark shift being eliminated by tuning the lattice laser to the “magic wavelength”^13,14^. Although the Doppler and recoil shifts can be reduced by confining the atoms in 1D optical lattices and probing along the lattice axis in the Lamb-Dicke and resolved sideband regimes^15–17^, for a finite temperature only a fraction of atoms occupy the motional ground state. The collisional shifts are directly related to this atomic temperature^18,19^. So the temperature as one of most important properties of a cold atom sample should be determined accurately. The conventional thermometry techniques of characterization of the cold atoms in optical lattices are time-of-flight (TOF) expansion imaging^20^ and thermometry based on the sideband spectrum (TSS). The former is realized by measuring the ballistic expansion of atomic clouds released from the trap at different times, while the latter is based on the sideband spectrum, for which the areas of spectrum of blue and red sidebands are different^16^. TOF is a routine method to determine temperatures of cold atoms in many laboratories, while TSS is developed to determine temperatures of cold ensembles in an optical lattice. These methods have a precision varying from a few percent to several tens of percent which depends on the experimental condition and the signal-to-noise ratio^16,20–25^.

In this paper, we show another thermometry technique based on the carrier spectrum (TCS), which is consistent with TOF and TSS. As is well known, TOF is no longer reliable due to low particle numbers or a lack of cycling transitions, and it needs two samples of cold atoms for probing different radii at different times, during which the cold atom samples are destroyed by the probe laser. TSS is based on the ratio of integrated sideband absorption and there is no contribution from the red sideband $({n}_{z}\to {n}_{z}-1)$ in the ground state (*n*_*z*_ = 0). We assume the atoms are not cold enough to degenerate, so they still satisfy the Boltzmann distribution which results in the expression as^16^

1 $$\frac{{\sigma }_{red}^{total}}{{\sigma }_{blue}^{total}}=\frac{\sum _{{n}_{z}=1}^{{N}_{z}}{e}^{-{E}_{{n}_{z}}/{k}_{B}{T}_{z}}}{\sum _{{n}_{z}=0}^{{N}_{z}}{e}^{-{E}_{{n}_{z}}/{k}_{B}{T}_{z}}}=1-\frac{{e}^{-{E}_{0}/{k}_{B}{T}_{z}}}{\sum _{{n}_{z}=0}^{{N}_{z}}{e}^{-{E}_{{n}_{z}}/{k}_{B}{T}_{z}}},$$

where ${\sigma }_{red,blue}^{total}$ is the area of the integrated sideband absorption, *k*_*B*_ is the Boltzmann constant. The longitudinal temperature *T*_*z*_ can be calculated directly from equation (1). However, the large relative uncertainty of determining the area of a vanishingly small red sideband and the low signal-to-noise ratio at lower temperature make it unreliable. Temperature measurement with TCS, on the other hand, can be determined from the expression^25^

2 $${T}_{C}\approx \frac{0.295{\Gamma }_{C}}{|\sqrt{\alpha \text{'}/\alpha }-1|}\frac{h}{{k}_{B}},$$

where *h* is the Planck constant, Γ_*C*_ is the full-width-at-half-maximum (FWHM) of the carrier line shape, $\alpha ^{\prime} /\alpha$ is the polarizability ratio of the excited and ground states which can be determined by the light shift due to the influence of the lattice laser. The broadened FWHM and the light shift caused by the lattice can be extrapolated from the carrier spectrum accurately due to the high signal-to-noise ratio of the carrier. This method is originally developed in ref.^25^ to map temperature of atoms or molecules in optical lattices via a differential spectroscopic light shift. TCS is limited to the narrow spectral lines of atoms or molecules confined tightly in optical lattices, which is suitable for the optical lattice clocks. We apply TCS to determine the temperature of cold ensembles in an ytterbium optical lattice clock. And we demonstrate the TCS method in an ytterbium optical lattice clock which is different from the experiment in ref.^25^. On the other hand, we show that the TCS method is an alternative method to map the temperature of cold ensembles in an optical lattice clock. From the accurately determined temperature it is possible to calculate the cold atom distributions and acquire such information as the light shift caused by thermal contributions and thermal line pulling. In this paper, we firstly present the clock transition spectrum including the carrier, the first-order and second-order sidebands spectrum. We then show the temperatures obtained by the three above mentioned methods. In conclusion the heating effects caused by the lattice laser are discussed.

## Results

### The clock transition spectrum

**Figure 1:**
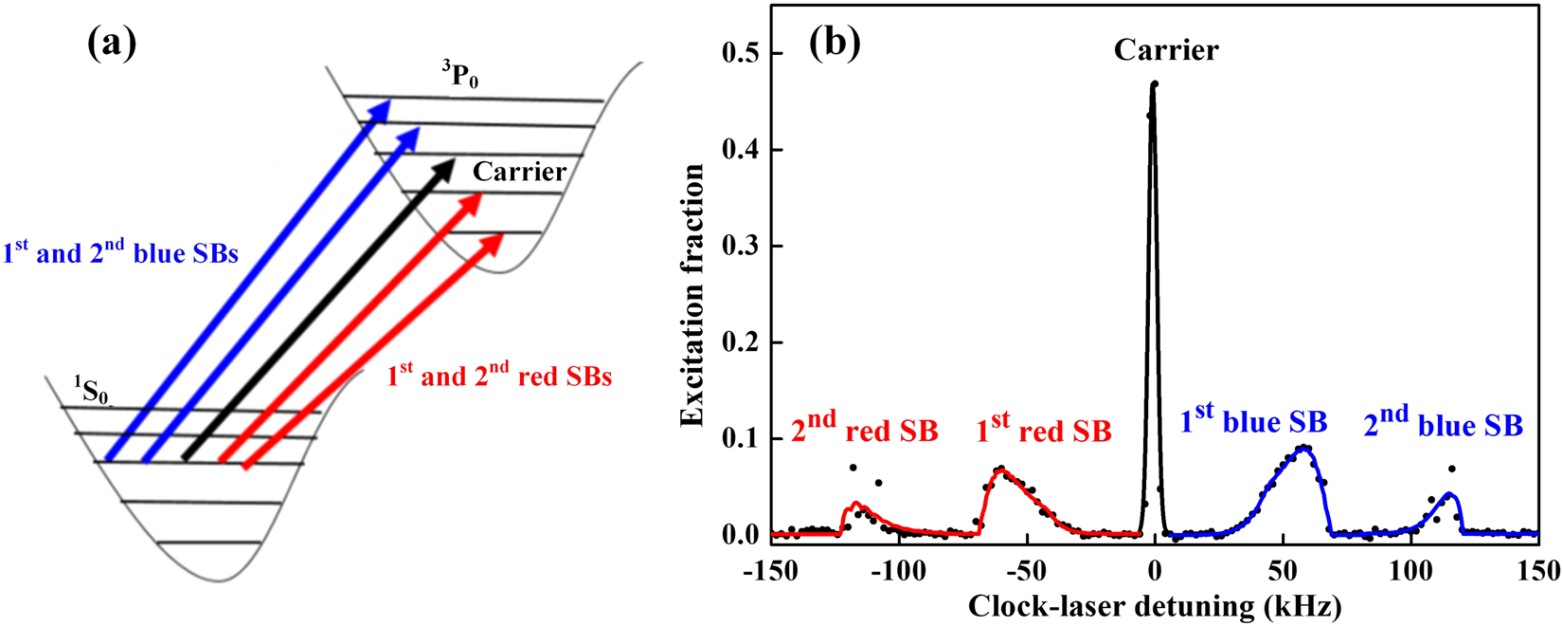
The clock transitions of different motional states in optical lattices. (**a**) Five dominant spectral features: the central carrier (black arrow) where the motional state is conserved; the first-order and second-order red sidebands (red arrows) where the motional state decreases; the first-order and second-order blue sidebands (blue arrows) where the motional state increases. (**b**) The spectrum including carrier, first-order sidebands and second-order sidebands.

After preparing ultracold ytterbium atoms and loading into the optical lattice (see Methods), the atoms are probed along the lattice axis in the Lamb-Dicke and resolved sideband regimes with the clock laser at 578 nm. As shown in Fig. 1(a), there are a series of possible transitions when the clock laser is tuned to a corresponding frequency. The transition for the same motional state is the carrier accompanied by the sideband transitions occurring at the different motional states^13,16,17^. Normally, the first-order sidebands are easily observed when the motional state is excited to the increased and decreased motional state by 1. The relative size of the sidebands is not only proportional to the Rabi frequency but has also other factors that influence the sideband intensities. The effective Rabi frequency of the *l*^th^ order sidebands is proportional to *η*^*l*^, where *η* is the Lamb-Dicke parameter that can be expressed as $\eta =\sqrt{{\nu }_{rec}^{p}/{f}_{z}}$. Here *f*_*z*_ is the trap frequency and ${\nu }_{rec}^{p}$ is the probe recoil frequency^26^. In the Lamb-Dicke regime *η* < 1, the sideband intensity shrinks drastically with increasing sideband order, which makes their detection problematic. The atoms in the optical lattice are not cold enough to degenerate and still satisfy the Boltzmann distribution, so there are still a number of atoms that populate a high motional state. This, in turn, means the second-order sidebands (motional state increases or decreases by 2) may be observed. Here we not only obtain the carrier and first-order sidebands spectrum, but also for the first time observe the second-order sidebands in the ytterbium optical lattice clock experiments, which is shown in Fig. 1(b). In ref.^16^, according to the harmonic approximation model, the first-order longitudinal energy gap is given as

3 $${\gamma }_{1}({n}_{z})={E}_{n+1}/h-{E}_{n}/h={f}_{z}-{\nu }_{rec}({n}_{z}+1)-{\nu }_{rec}\frac{{\nu }_{r}}{{\nu }_{z}}({n}_{x}+{n}_{y}+1),$$

and the first-order blue sideband line shape is given as

4 $${\sigma }_{Fblue}(\delta )\propto \sum _{{n}_{z}=0}^{{N}_{z}}{e}^{-\frac{{E}_{{n}_{z}}}{{k}_{B}{T}_{z}}}(1-\delta /{\tilde{\gamma }}_{1}({n}_{z})){e}^{-{\alpha }_{1}(1-\delta /{\tilde{\gamma }}_{1}({n}_{z}))}{\rm{\Theta }}[{\tilde{\gamma }}_{1}({n}_{z})-\delta ],$$

where ${\tilde{\gamma }}_{1}({n}_{z})={f}_{z}-{\nu }_{rec}({n}_{z}+1),\,{\alpha }_{1}=\frac{{\tilde{\gamma }}_{1}({n}_{z})}{{\nu }_{rec}}\frac{h{f}_{z}}{{k}_{B}{T}_{r}}$, and Θ is the Heaviside function.

We can write the second-order longitudinal energy gap as

5 $${\gamma }_{2}={E}_{n+2}/h-{E}_{n}/h=2{f}_{z}-{\nu }_{rec}(2{n}_{z}+3)-2{\nu }_{rec}\frac{{\nu }_{r}}{{f}_{z}}({n}_{x}+{n}_{y}+1),$$

and the second-order blue sideband line shape as

6 $${\sigma }_{Sblue}(\delta )\propto \sum _{{n}_{z}=0}^{{N}_{z}}{e}^{-\frac{{E}_{{n}_{z}}}{{k}_{B}{T}_{z}}}(1-\delta /{\tilde{\gamma }}_{2}({n}_{z})){e}^{-{\alpha }_{2}(1-\delta /{\tilde{\gamma }}_{2}({n}_{z}))}{\rm{\Theta }}[{\tilde{\gamma }}_{2}({n}_{z})-\delta ],$$

where ${\tilde{\gamma }}_{2}({n}_{z})=2{f}_{z}-{\nu }_{rec}(2{n}_{z}+3)$ and ${\alpha }_{2}=\frac{{\tilde{\gamma }}_{2}({n}_{z})}{2{\nu }_{rec}}\frac{h{f}_{z}}{{k}_{B}{T}_{r}}$. The expressions indicate that the sidebands have the same line shape and the sideband spectrum in Fig. 1(b) can be fitted by them. According to the model, the longitudinal energy gap between different motional states is dependent not only on the longitudinal but also the transverse motional states. We determine that the energy gap shrinks as the motional state increases, which means that the frequency (122 kHz in Fig. 1(b)) of the sharp edge in the second-order sideband is lower than double of that (69 kHz in Fig. 1(b)) in the first-order sideband.

**Figure 2:**
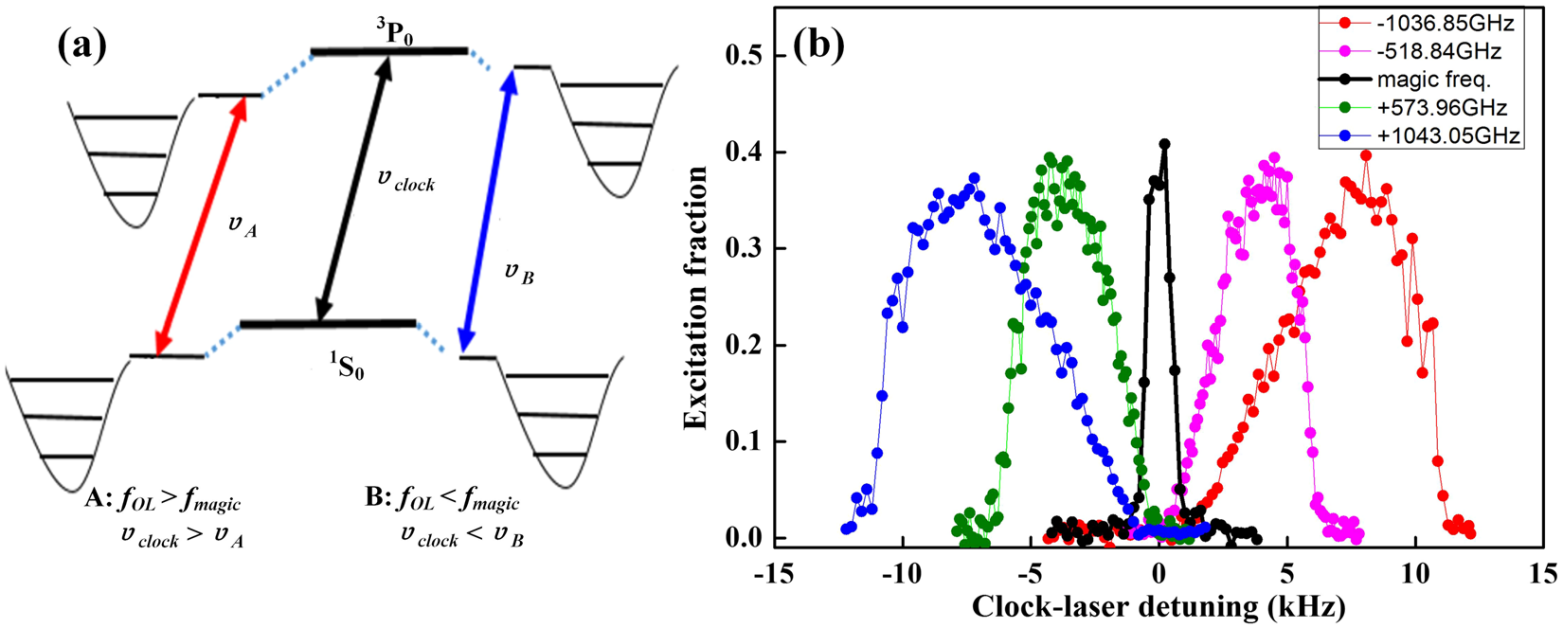
The carrier spectrum. (**a**) The central carrier transitions for different lattice frequencies. Far away from the magic frequency, the line is broadened and shifted due to the ac Stark effect. (**b**) The carrier spectra for different lattice frequencies, where the line shape is symmetric and narrow at the magic frequency accompanied by the asymmetric and broadened line shapes at non-magic frequencies.

The optical lattice clock is operated at the magic wavelength, where the polarizability of the excited and ground states is equal and the ac Stark shift is eliminated. When operating the optical lattice at a non-magic wavelength, the polarizability of the excited and ground state is no longer the same^13,17,27,28^, accordingly the lattice trap depth of the excited and ground state is different due to the linear relation to the polarizability. The different polarizabilities lead to the ac Stark shift which can be expressed as ${\rm{\Delta }}\nu =-\,\frac{1}{4}(\alpha ^{\prime} -\alpha )I-\frac{1}{64}{\rm{\Delta }}\gamma {I}^{2}+\cdots$. For ^171^Yb in optical lattices, the polarizability of the excited state is larger than the polarizability of the ground state $(\alpha ^{\prime}  > \alpha )$ when the lattice frequency is higher than the magic frequency and the inverse relation ($(\alpha ^{\prime}  < \alpha )$) is valid for the case when the lattice frequency is lower than the magic frequency^14^. Considering the polarizability as the dominant term, the light shift is positive for ${f}_{OL} < {f}_{magic}$ and negative for ${f}_{OL} > {f}_{magic}$, which is shown in Fig. 2(a).

Due to different lattice trap depths of the excited and ground state at a non-magic wavelength, the carrier transition frequency of the *n*_*z*_ motional state deviates from the nominal clock frequency *ν*_0_ by a value of *n*_*z*_*dν*. Here *dν* is the difference between the excited state and ground state trap frequencies. With the Boltzmann distribution of cold atoms in the lattice, the spectrum of the carrier can be considered as a superposition of all the motional states and the line shape of a single transition between two motional states is a Lorentzian form with the weight of the occupation probability ${p}_{n+1}/{p}_{n}={e}^{-h{f}_{z}/{k}_{B}T}={f}_{B}$. Also the light shift caused by the lattice laser includes two parts of the thermal and nonthermal light shift and can be expressed^25^ as $W\approx (1-\frac{\alpha ^{\prime} }{\alpha }){U}_{0}+3(\sqrt{\frac{\alpha ^{\prime} }{\alpha }}-1){k}_{B}T$. The nonthermal light shift $(1-\frac{\alpha ^{\prime} }{\alpha }){U}_{0}$ has no correlation with the temperature, and thus has the same value for all motional states. The thermal light shift $3(\sqrt{\frac{\alpha ^{\prime} }{\alpha }}-1){k}_{B}T$ is linear with the temperature. The atoms with lower temperature populate lower motional states and experience a smaller light shift. The thermal and nonthermal light shifts are reversed in sign. With the Boltzmann distribution of atoms populating different motional states, the carrier line shape is asymmetrical and broadened. The steep edge always faces the light shift direction as shown in Fig. 2(b).

### Thermometry

We measure the temperature of cold atoms in the lattice with a trap depth characterized by the recoil energy, ${E}_{R}={h}^{2}/(2m{\lambda }_{L}^{2})$, imparted to an atom of mass *m* by a lattice photon with wavelength *λ*_*L*_. For the thermometry experiments, the trap depth *U*_0_ = 450*E*_*R*_ is obtained from the spectrum including the carrier and first-order sidebands.

**Figure 3:**
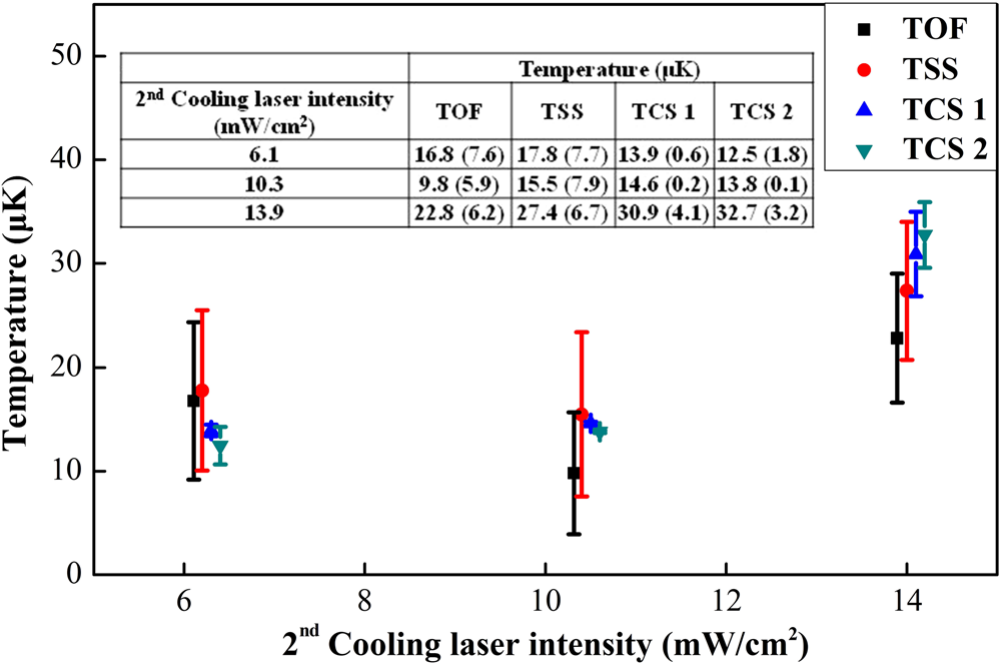
The temperatures measured by three different methods. Temperatures measured by the three above mentioned methods, as a function of the 556 nm laser intensity (the second cooling laser). TCS 1 and TCS 2 are the measured temperatures at the lattice frequency of 395,372.29(0.01) GHz and 394,279.49(0.01) GHz. For a clear display, an offset is added to the 2^nd^ cooling laser intensity of different methods. The inset sheet shows the temperature values. The error bar represents the 1σ uncertainty.

The cold atoms are imaged by an intensified CCD camera (ICCD, Andor iStar 334 T) at 5 ms and 10 ms after the lattice laser is shut and the atom clouds emit fluorescent light when the ${}^{1}\,{S}_{0}\to {}^{1}\,{P}_{1}$ transition at 399 nm is excited. The temperatures can be calculated according to

7 $$T=\frac{m}{2{k}_{B}}[\frac{{a}^{2}({t}_{2})-{a}^{2}({t}_{1})}{{t}_{2}^{2}-{t}_{1}^{2}}],$$

where *t*_1_, *t*_2_ are the times after the lattice laser is shut, *a*(*t*_1_), *a*(*t*_2_) are the expansion radii of the atom clouds released from the optical lattice trap at time of *t*_1_ and *t*_2_, which can be directly read out from the images. The temperatures are plotted for three different intensities of 556 nm laser (the second cooling laser) in Fig. 3, where each dot is averaged over four independent measurements. The temperatures obtained by TSS in Fig. 3 are measured in the vicinity of the magic wavelength^8,29,30^ as 394,798.33(0.01) GHz. The unbroadened carrier linewidth is about 6.9 Hz, approaching the Fourier-limited linewidth with a 150 ms interrogation time^31^. The temperatures in Fig. 3 are acquired from the first-order sideband spectrum. The temperatures also can be determined by the area ratio of the second-order sidebands, but the smaller excited state fraction and lower signal-to-noise ratio hinder the precision, and the visible second-order red sideband $({n}_{z}\to {n}_{z}-2)$ only appears when some atoms occupy the vibrational states of *n*_*z*_ ≥ 2, which means a higher temperature^32–34^. Although only the longitudinal temperature is determined from the first-order sideband spectrum and equation (1), the spectroscopic line shape is determined by the coupling between the longitudinal and transverse degrees of freedom due to the Gaussian lattice beam profile, which results in dependency of the longitudinal transition frequency on the transverse motional state. By fitting the sideband line shape with equation (4), the transverse temperature can be extracted from the same data used for TSS. The data show a close value of the longitudinal and transverse temperatures, and there is no apparent increase in transverse temperature.

**Figure 4:**
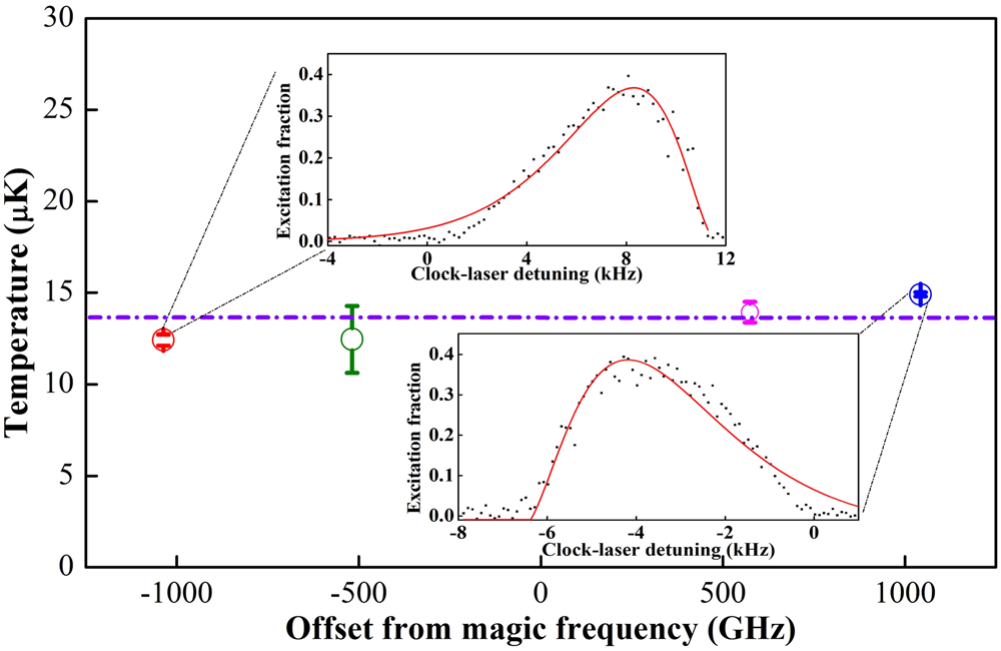
Temperature measured by TCS. The temperatures measured by TCS at different lattice frequencies. Each circle represents a value averaged over four measurements. The purple line represents the average temperature value of the measurements. The insets are the fittings of the carrier spectra at the lattice frequency of 395,372.29(0.01) GHz and 393,761.48(0.01) GHz. The error bar represents the 1σ uncertainty.

The temperatures obtained by TCS in Fig. 3 are determined from the carrier spectrum (Fig. 2(b)) and equation (2). The two unknown parameters of equation (2) Γ_*C*_ and *α*′/*α* can be determined from the carrier spectrum as shown in Fig. 2(b), for which the FWHM can be derived directly. The polarizability ratio of the excited and ground states *α*′/*α* can be extrapolated by the expression of $\alpha ^{\prime} /\alpha =1-2{W}_{0}/(m{\lambda }_{L}^{2}{f}_{z}^{2})$. *W*_0_ is the nonthermal light shift caused by the lattice laser^25^. It follows from equation (2) that for the case of operating the lattice at the magic frequency, the temperature of the atoms is infinite due to the polarizabilities of the excited and ground states being equal. This conclusion is obviously in conflict with reality, so the lattice laser has to be tuned far away from the magic frequency to obtain the light-shifted and broadened spectrum. The light shift value can be obtained from the fitting parameters (the insets of Fig. 4 are the fittings of the carrier spectra) which can yield the value of *α*′/*α* (see Methods). The theoretical spectroscopic line shape of equation (8) (see Methods) is a continuous approximation of the discrete expression. Due to the narrow spectral line of carrier transition from discrete motional states, there is a discrepancy between the measured line shapes and the theoretical fits.

As shown in Fig. 4, the temperatures measured by TCS have no clear dependence on the lattice frequency, so they may represent the temperature at the magic frequency. The purple line in Fig. 4 indicates the average temperature of the four circles. Although we can determine the temperature at any frequency except the magic frequency for a far-off-resonance trap, in consideration of the error bars in Fig. 4, it is better to measure the temperature at the frequency further away from the magic frequency. In other words, the larger light shift makes the measurement more accurate. As shown in Fig. 3, the three methods are in good agreement, even though TOF and TCS yield 3D temperatures and TSS only yields the longitudinal temperature. In our experiment, TOF and TSS have about 40% uncertainties while TCS has an uncertainty below 10%. The error bars for TCS are smaller than TOF and TSS by roughly a factor of 5.

### The parametric heating effects caused by the lattice

**Figure 5:**
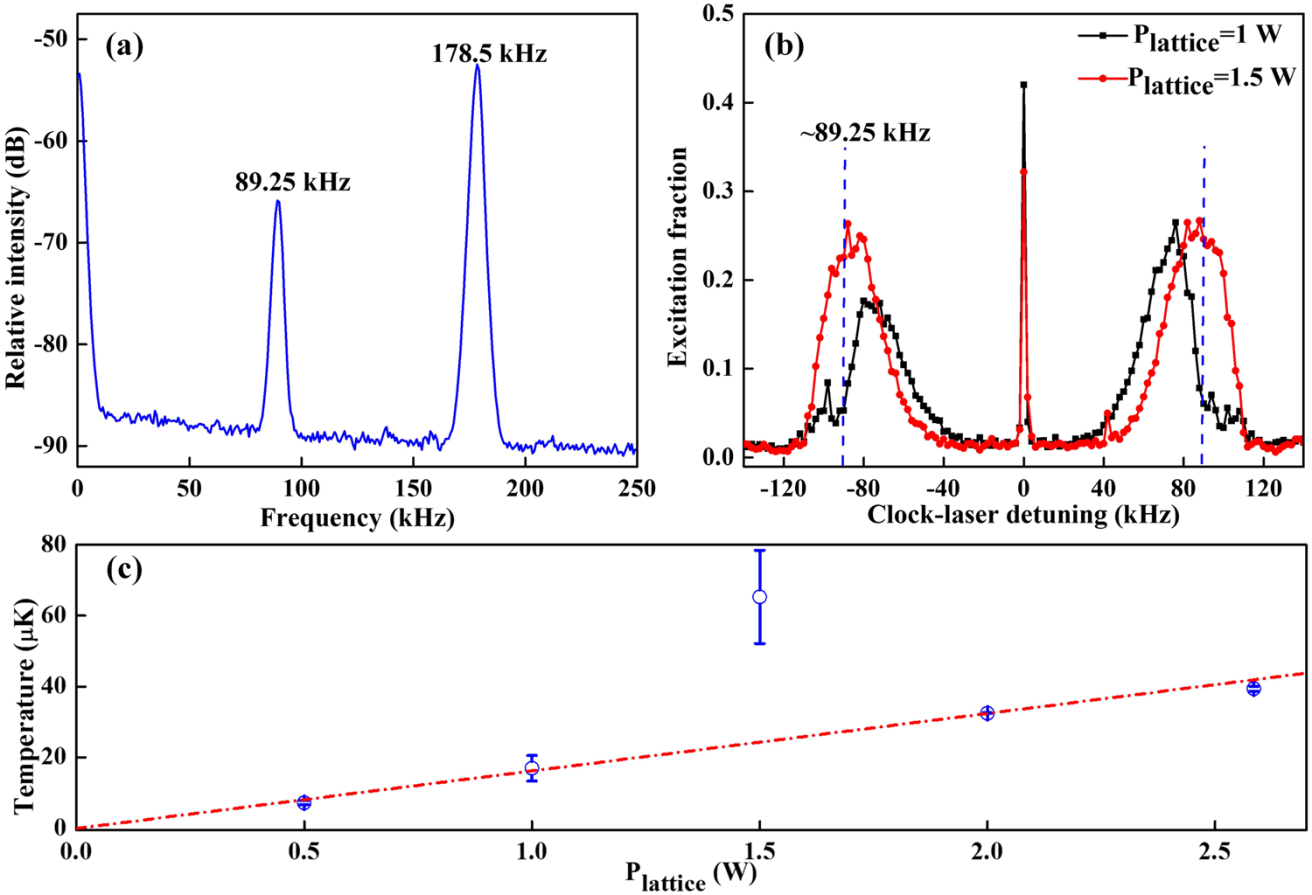
The heating and the dependence of temperature on the lattice power. (**a**) Intensity noise power spectrum and most of the intensity noise are distributed at frequencies of 178.5 kHz and 89.25 kHz. (**b**) The spectrum of atomic excitation at different lattice powers of 1.5 W and 1 W. The blue lines are at the detuning of ±89.25 kHz. (**c**) The dependence of the temperature on the lattice power. The error bar represents the 1σ uncertainty.

The ultracold atoms in the optical lattice will be heated due to the spontaneous scattering of trap laser photons^35^ and the technical heating^36,37^ caused by the intensity fluctuations and pointing instabilities of the trapping laser beams. Figure 5(c) shows the dependence of temperature on the lattice laser power. It indicates that the temperature increases with increasing lattice power, which can be explained by the proportional relation of the photon scattering rate to the laser intensity as ${R}_{s}\approx \frac{{U}_{0}{\rm{\Gamma }}}{\hslash {\rm{\Delta }}}$. The heating caused by intensity fluctuations is also in the linear relation with the trap potential as ${{\rm{\Gamma }}}_{\varepsilon }\equiv {\pi }^{2}{f}^{2}{S}_{\varepsilon }(2f)$. The increased energy caused by heating can be expressed by $\langle \dot{E}\rangle =2{R}_{s}{E}_{R}$ and $\langle \dot{E}\rangle ={{\rm{\Gamma }}}_{\varepsilon }\langle E\rangle$. Here Γ is the natural linewidth, Δ is the detuning of the lattice laser, *S*_*ε*_ is the power spectrum of the fractional intensity noise, *E*_*R*_ is the recoil energy and *f* is the trap energy gap frequency. In Fig. 5(c) the temperature at the lattice laser power of 1.5 W is high and does not follow the linear approximation. This can be explained by the relationship between the heating rate and the lattice laser intensity noise, which indicates that the heating rate is proportional to the noise power density at the second harmonic of the trap energy gap frequency. Figure 5(a) shows the intensity noise power spectrum of our lattice laser (Ti:Sa laser). In the experiment, most of the lattice laser intensity noise is distributed at frequencies of 178.5 kHz and 89.25 kHz. Therefore, the cold atoms in the lattice will be heated and the temperature will increase at the trap energy gap frequencies of 89.25 kHz and 49.125 kHz. Figure 5(b) shows the clock transition spectra at the lattice laser power of 1.5 W and 1 W, which yields the trap frequencies of 110 kHz and 90 kHz. Due to the Gaussian intensity distribution of the lattice laser and the coupling of the longitudinal and the transverse degrees of freedom, the frequency of the energy gap^16,38^ is expressed as $f\approx {f}_{z}{e}^{-{r}^{2}/{w}_{0}^{2}}-{E}_{R}(n+1)/h$. The heating rate of a single atom has dependence linearly on the noise power density at the second harmonic of the trap energy gap frequency. The blue dashed lines of Fig. 5(c) indicate the clock laser detuning of ±89.25 kHz, which correspond to the values of the energy gap frequencies. At these frequencies a larger fraction of atoms are excited in the lattice of 1.5 W than 1 W. So the increased temperature occurs at the power of 1.5 W rather than 1 W and the trap depth must be far detuned from the peak intensity noise frequency when the optical lattice clock is in operation.

## Discussion

The temperature of the ultracold atoms, which has a direct impact on the uncertainty and instability of atomic clocks, should be characterized at a high precision level. The carrier spectrum provides important information on the state of the system and the carrier spectrum at a non-magic wavelength can yield the temperature accurately. The heating caused by the lattice laser intensity fluctuation is observed in our experiment. For an optimal clock operation, the trap frequency should be set far from half the peak intensity noise frequency of the lattice laser.

## Methods

### Sample preparation and spectrum probe

**Figure 6:**
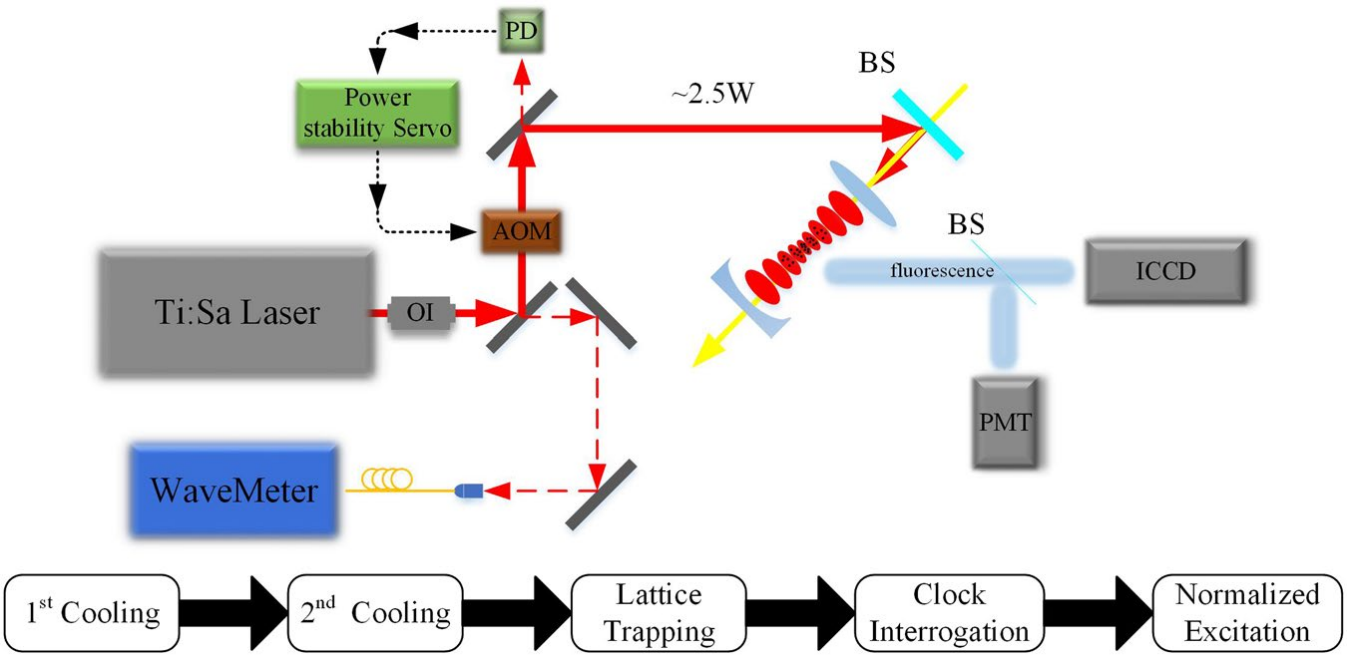
Simplified experimental setup, and the sequence of the sample preparation and spectrum probe. ICCD: intensified CCD camera; PMT: photomultiplier; PD: photo-detector; AOM: acousto-optic modulator; BS: beam splitter; OI: optical isolator.

Figure 6 shows the simplified experimental setup, and the sequence of sample preparation and spectrum probe. The hot ytterbium atoms is first cooled to about 1 mK using a Zeeman slower, a 2D optical molasses and a 3D magneto-optical trap (MOT) on the ${}^{1}\,{S}_{0}\to {}^{1}\,{P}_{1}$ 29 MHz transition at 399 nm. Then the atoms are further cooled to a few μK with the 3D MOT operating on the ${}^{1}\,{S}_{0}\to {}^{3}\,{P}_{1}$ 182 kHz transition at 556 nm. The atoms are subsequently loaded into the optical lattice where the clock transition is interrogated with a pulse at 578 nm. The spectrum is logged by the normalized detection with the repumping lasers on the ${}^{3}\,{P}_{0}\to {}^{3}\,{S}_{1}$ transition at 649 nm and the ${}^{3}\,{P}_{2}\to {}^{3}\,{S}_{1}$ transition at 770 nm. More details and the experimental setup can be found in our previous works^27,39–41^. The maximum of the lattice laser power is about 2.5 W and the power is stabilized by using an acousto-optic modulator (AOM). Its frequency is locked to the build-in cavity and can be measured by a wavemeter with the 10-MHz uncertainty.

### Thermometry based on carrier spectrum

The line shape of the carrier can be expressed^25^ as

8 $$p(u)=\frac{1}{2}{u}^{2}{e}^{-u},$$

where $u(\delta {E}_{i})=\frac{\delta {E}_{i}}{{k}_{B}T(\sqrt{\frac{\alpha ^{\prime} }{\alpha }}-1)}\ge 0$ is a dimensionless function of the Boltzmann distribution, *δE*_*i*_ is a differential light shift. The equation (2) is deduced from equation (8) as its FWHM is 3.395 and expressed by the form of ${{\rm{\Gamma }}}_{u}=3.395=\frac{\delta {E}_{i}}{{k}_{B}T(\sqrt{\frac{\alpha ^{\prime} }{\alpha }}-1)}=\frac{h{{\rm{\Gamma }}}_{C}}{{k}_{B}T(\sqrt{\frac{\alpha ^{\prime} }{\alpha }}-)1}$. The experimental data in Fig. 3(b) is fitted by the line shape function as $y={y}_{0}+a{(b|x|-c)}^{2}{e}^{-(b|x|-c)}$. The parameter *u* is zero as *δE*_*i*_ = 0, which corresponds to zero light shifts. So the nonthermal light shift is the zero point of the line shape function. The fitting parameters yield the nonthermal light shift as *W*_0_/*h* = *c*/*b*.
